# Wide-field mid-infrared hyperspectral imaging of adhesives using a bolometer camera

**DOI:** 10.1038/s41598-017-11994-4

**Published:** 2017-09-29

**Authors:** Shigeru Sugawara, Yoshihiko Nakayama, Hideya Taniguchi, Ichiro Ishimaru

**Affiliations:** 1 National Research Institute of Police Science, Kashiwa, Japan; 2 Aoi Electronics co., ltd., Takamatsu, Japan; 3 Kagawa University, Takamatsu, Japan

## Abstract

By combining a bolometer detector with an imaging-type interferometer, an inexpensive, easy-to-handle wide-field mid-infrared hyperspectral imaging apparatus was produced. We measured the distributions of four types of thin adhesive layers on an aluminium plate and analysed the results using correlation coefficients to visualise the distribution of various adhesives that cannot be discerned by the naked eye or conventional methods such as visible/near-infrared spectroscopic/fluorescent photography. The measurement wavelength range, obtained spectrum’s wavenumber resolution, and measurement time was 8–14 μm, about 9 cm^−1^, and about 30 s, respectively. Using conventional methods, adhesives could not be distinguished from the others. By using this method, we found that adhesives could be precisely distinguished by setting an appropriate threshold value for the correlation coefficient. Thus, our approach can accurately measure the spatial distribution of different types of adhesive that cannot be discriminated by conventional methods.

## Introduction

Efficient and secure detection of trace evidence in the form of residues at crime scenes, such as fingerprints, blood marks, fine objects and document tampering, is an important issue in forensic science. Early detection of deterioration or pollution on cultural property is also an important issue in the science of cultural properties. One effective method for inspecting these samples, which is difficult to do with the naked eye, is to use special cameras. Conventionally, such samples have been inspected using spectroscopic cameras and fluorescent cameras in the visible and near-infrared wavelength range 0.4–1.0 μm; however, some samples are difficult to detect with these conventional methods.

To overcome the limitations of conventional spectroscopic and fluorescent cameras, we have developed a method for inspecting the aforementioned difficult samples using mid-infrared hyperspectral imaging, with wavelengths in the range 8–14 µm, which are longer than those used in conventional methods. The reason for choosing this wavelength band is its high discrimination ability for organic materials. However, with conventional microscopic mid-infrared hyperspectral imaging apparatus, we cannot measure the macroscopic-sized samples that are treated in forensic science and cultural properties science^1,2^. Wide-field mid-infrared hyperspectral imaging apparatus employing mercury cadmium telluride (MCT) detectors could be used^3–6^, but they are very expensive and difficult to handle legally because MCT detectors are a type of military equipment, thus making this approach difficult to use for daily work in forensic science and cultural properties science.

Bolometer detectors are cheaper than MCT detectors by about two orders of magnitude and are easy to handle legally. Therefore, if a bolometer detector were used, it is expected that a mid-infrared hyperspectral imaging apparatus with high versatility could be realised at low cost. However, bolometer detectors are said to be about two orders of magnitude lower in sensitivity than MCT detectors. Therefore, measurements will be difficult if the bolometer is combined with an ordinary spectroscope. By combining a bolometer detector with an imaging-type interferometer, which has high light-utilisation efficiency, we were able to produce a measurement apparatus that is inexpensive and easy to handle. Since the imaging-type interferometers are more compact than the Michelson-type interferometers, the whole apparatus also became compact and easy to handle^7–11^.

To test our new apparatus, we attempted to measure samples that are encountered in forensic science and cultural properties science. It is obvious that more samples can be measured by using an MCT detector rather than a bolometer, but here we are dedicated to bolometer detectors and we have studied what kinds of samples can be measured. As a result, it was found that organic material on highly reflective objects such as metal surfaces can be successfully measured^12^. Therefore, we measured the distribution of thin adhesive layers on an aluminium plate and analysed the results by statistical processing to visualise the distribution of various adhesives that cannot be discerned by the naked eye.

## Methods

**Figure 1:**
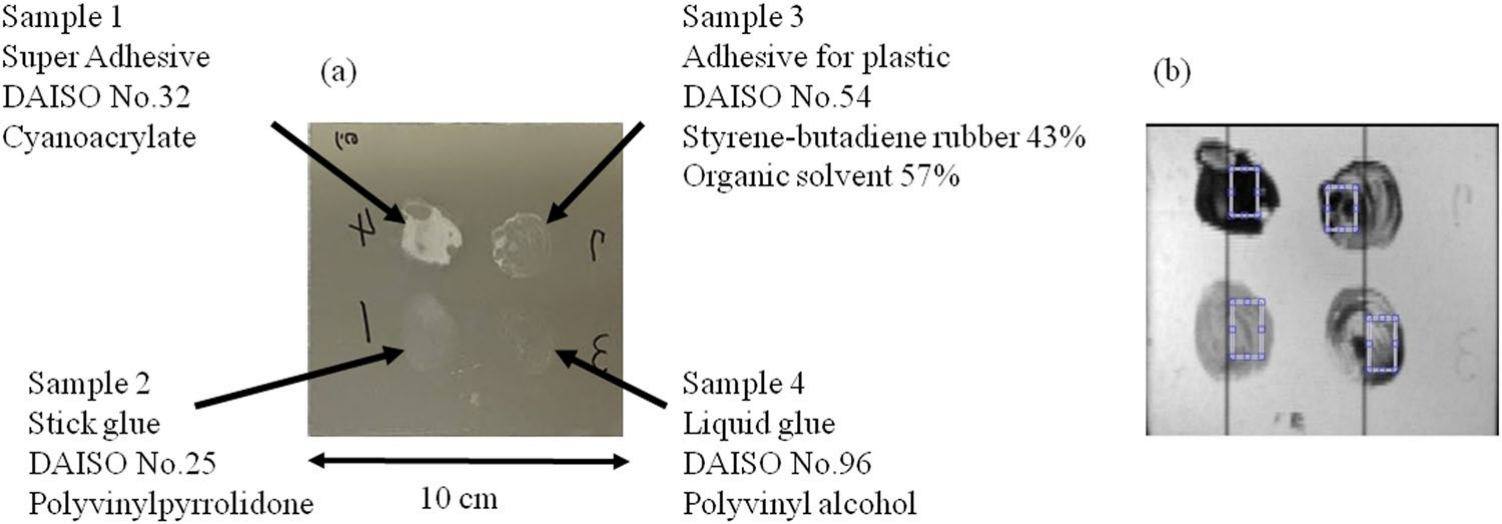
(**a**) Samples used in this study; four types of adhesive were spread on a 10 cm × 10 cm aluminum plate. (**b**) Rectangular area used to calculate the representative spectrum of each sample.

**Figure 2:**
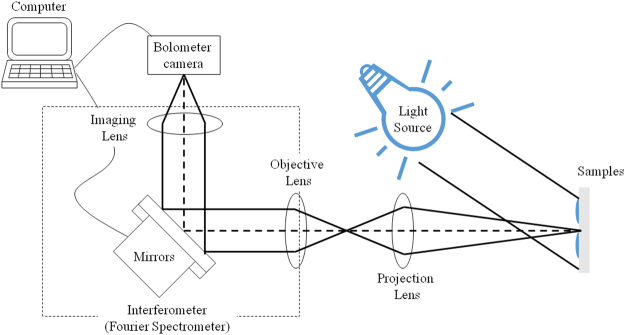
Optical system of the mid-infrared hyperspectral imaging device.

Four types of adhesives were coated on a 10 cm × 10 cm aluminium plate (Fig. 1(a)). The mid-infrared hyperspectral imaging apparatus (Fig. 2) was created by combining an imaging interferometer with a bolometer camera (Nippon Avionics Co., Ltd. C100 V)^6^. The spectra of the sample were also measured by a commercially available FTIR apparatus (Spotlight300, Perkin Elmer co., ltd.).

The measurement wavelength range was 8–14 μm, the wavenumber resolution of the obtained spectrum was about 9 cm^−1^, the measurement time was about 30 seconds, and the temperature resolution of the camera was 50 mK. A 300 °C black body with a size of 15 cm × 15 cm was used as the light source. The measurement area can be freely changed by the position of the projection lens, but in this experiment an approximately 10 cm × 10 cm square was measured. In order to compare the measurement result with the conventional method, visible/near-infrared spectroscopic/fluorescence photography over the wavelength range 0.4–1.0 µm was also measured using a Foster + Freeman VSC − 6000/HS.

The obtained interferograms were multiplied by Blackman’s window function and then Fourier transformed to obtain a spectrum for each pixel. In the imaging interferometer, the amount of phase shift varies depending on the field angle from the camera, so the spectral data were corrected according to previously published methods^13^. In order to align the scales of the wavenumber axes of all pixels, data were interpolated using a spline function. In order to suppress the influence of the spatial unevenness of the amount of light from the light source, the absorbance was calculated for each pixel and wavelength using the reflectance when nothing was deposited on the aluminum plate as the background “equation (1)”. In order to suppress the noise in the measurement data, two-dimensional Gaussian spatial filtering with a standard deviation of 3 was performed for each wavelength.

**Figure 3:**
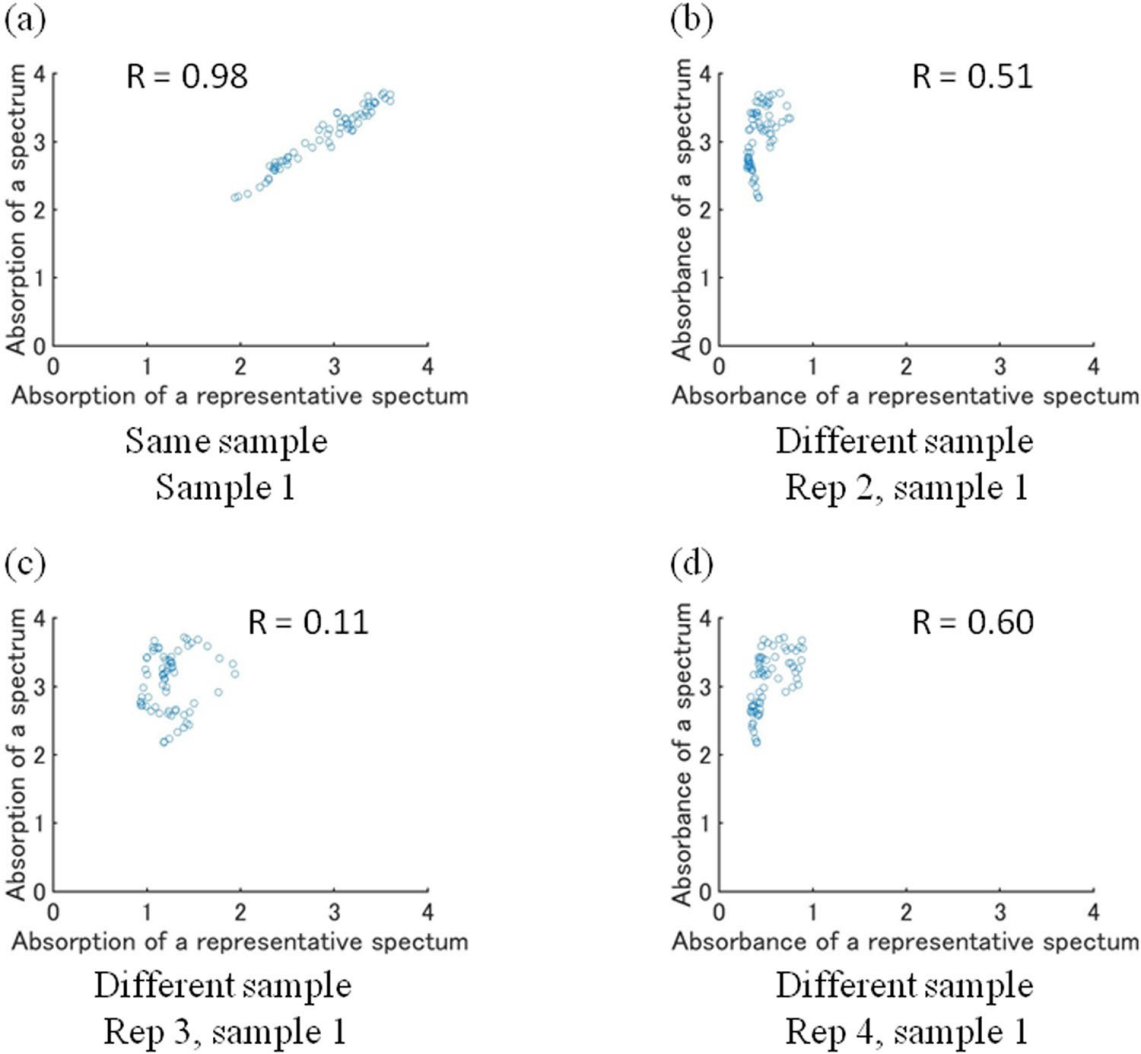
Plot of the relationship between the absorbance taken from the representative spectrum of a sample and the absorbance taken from the spectrum of a certain pixel at the same wavelength; (**a**) for the same sample and (**b**), (**c**), (﻿**d**) for different samples.

In order to visualise the distribution of each sample on the aluminium plate, the hyperspectral imaging data were analysed using correlation coefficients. First, rectangular regions were selected from the pixels at which only one sample was present (Fig. 1(b)), and the absorbance spectra of all of the points in the region were averaged to obtain a representative spectrum for each sample. Next, as shown in Fig. 3, the absorbance at each wavelength from the spectrum of a single pixel was plotted against the absorbance from the representative spectrum of each sample, and the correlation coefficient was calculated to evaluate its linearity “equation (2)”. For example, in the case of spectra from the same sample, as the absorbance on the horizontal axis increases, the absorbance on the vertical axis also increases, so the plots tend to be straight lines, as shown in Fig. 3a. However, even with the same sample, the thickness of the sample varies depending on the location, so the inclination of the straight line is not necessarily 1. On the other hand, in the case that single-pixel and reference spectra are from different samples, the absorbance on the vertical axis does not necessarily vary with wavelength in the same manner as the absorbance on the horizontal axis, so the plot is not a straight line, as shown in Fig. 3b. As described above, the correlation coefficients between the representative spectrum for each sample and the spectra from each pixel were calculated, and the magnitudes were used as the brightness values for the respective pixels, thus enabling the spatial distribution of each sample to be examined. In order to make the differences between correlation coefficients easier to discern by human vision, the brightness of the image was set to be proportional to the square of the correlation coefficient.

R: correlation coefficient, t:wavelength, N:Number of measurement points of wavelength,

f(t): representative spectra, g(t):spectrum of a point, f0 and g0:average of absorbance of the spectra.

1 $${\rm{Absorbance}}=-{\rm{In}}(\frac{{\rm{sample}}\,{\rm{reflectance}}}{{\rm{background}}\,{\rm{reflectance}}})$$

2 $${\rm{R}}=\frac{\frac{1}{N}{\sum }_{t=1}^{N}(f(t)-{f}_{0})(g(t)-{g}_{0})}{\sqrt{\frac{1}{N}{\sum }_{t=1}^{N}{(f(t)-{f}_{0})}^{2}}\sqrt{\frac{1}{N}{\sum }_{t=1}^{N}{(g(t)-{g}_{0})}^{2}}}$$

## Results

**Figure 4:**
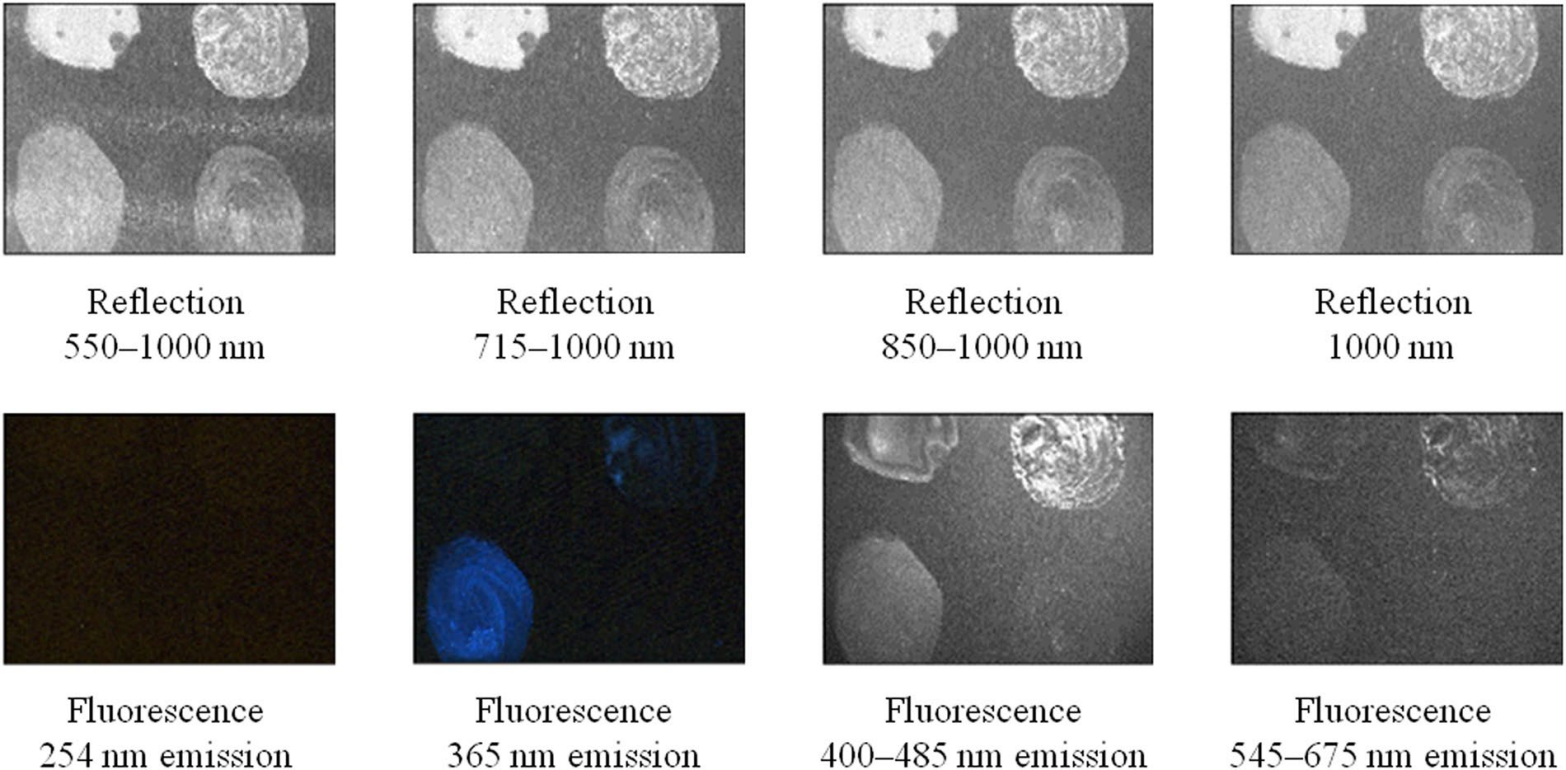
Results obtained by measuring the sample using the conventional visible/near-infrared spectroscopic/fluorescent photography method.

Figure 4 shows results measured by the conventional visible/near-infrared spectroscopic/fluorescent photography method. Although the upper and lower portions of the picture were slightly cropped due to restrictions of the apparatus, it was possible to determine the characteristics of each sample. In the conventional method, 63 kinds of measurement were performed by changing the transmission wavelength of two bandpass filters, one of which exists between the light source and the sample and the other between the sample and the camera, or by changing the type of light source from a halogen lamp to an ultraviolet lamp. Due to space limitations, only 8 representative results are shown here. Since sample 2 emitted fluorescence and sample 4 slightly emitted fluorescence when irradiated by ultraviolet light of wavelength 365 nm, sample 1 and sample 4 could not be distinguished by the conventional method. Sample 2 and sample 3 also could not be distinguished by the conventional method because of the same reason.

**Figure 5:**
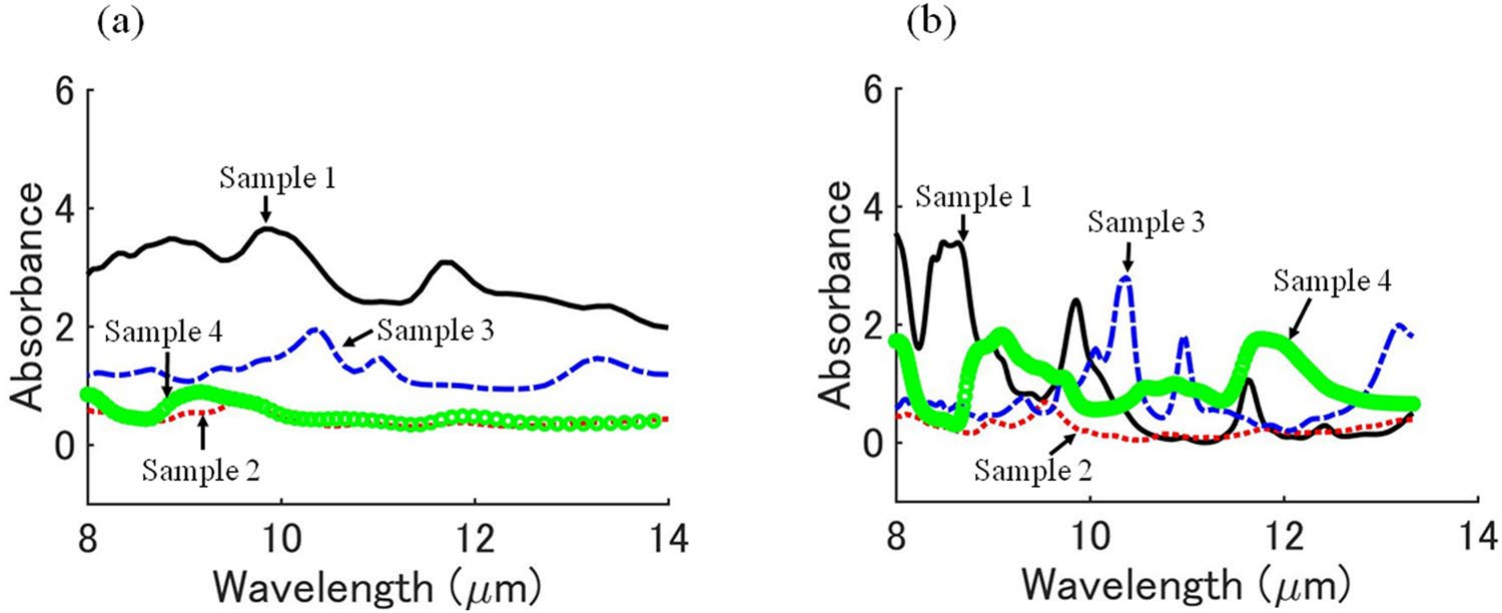
(**a**) Representative spectra of each sample. (**b**) Spectra measured by a commercial FTIR apparatus.

Representative spectra of each sample are shown in Fig. 5(a). This is the average spectrum of all of the spectra present in the preselected region that was selected for each sample. The spectra of the sample measured by a commercially available FTIR are shown in Fig. 5(b). In the correlation coefficient analysis, the correlation coefficients between each representative spectrum and the spectra of each pixel were calculated for each sample. Samples 2 and 4 had similar-shaped spectra except for wavelengths around 8–9 μm, and the other samples had relatively more unique spectra.

**Figure 6:**
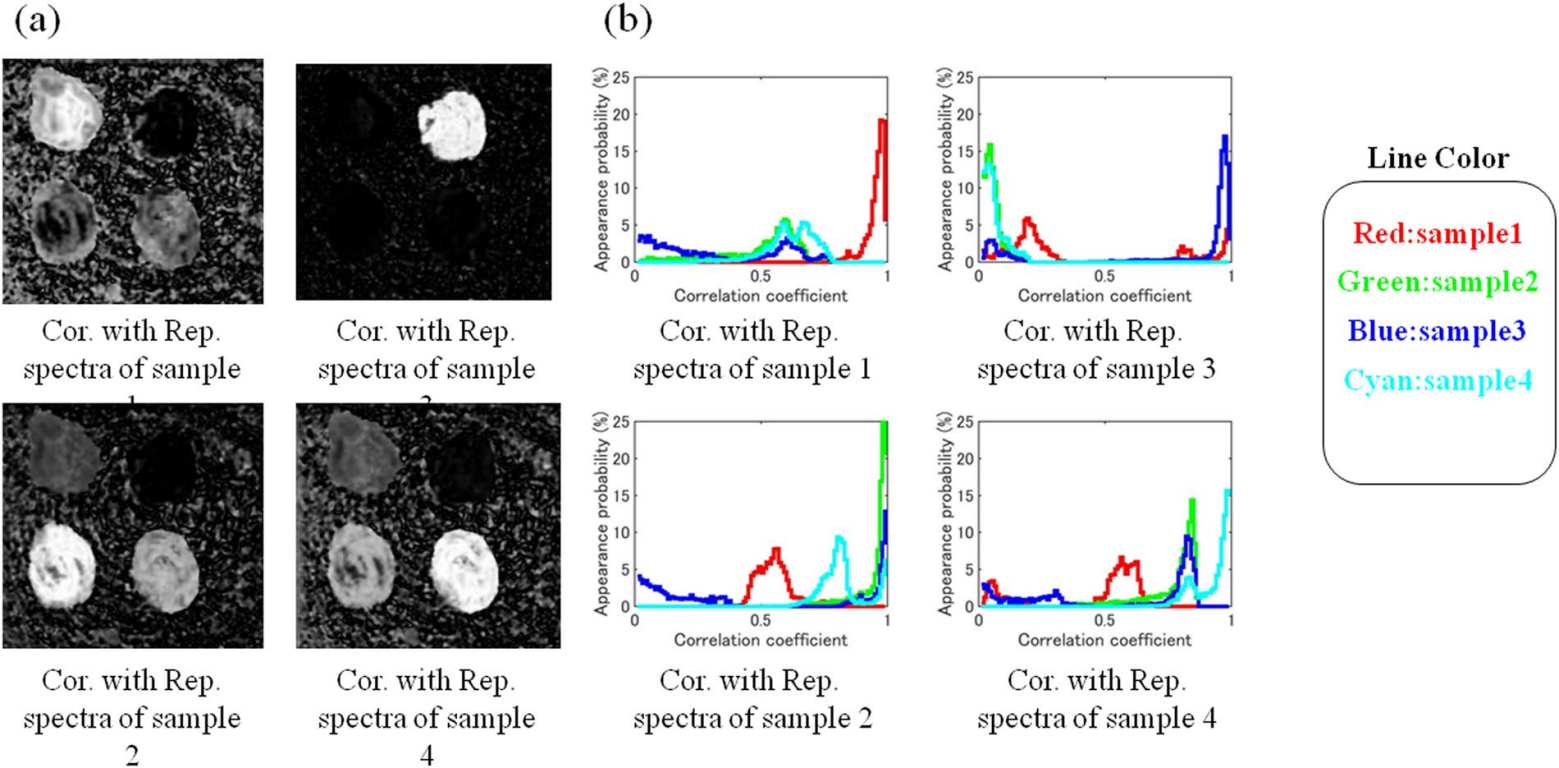
(**a**) Estimated distribution of each sample obtained by correlation coefficient analysis. The same hyperspectral data was processed with different representative spectra leading to images highlighting different samples. (**b**) Frequency of appearance of correlation coefficient between the representative spectrum of each adhesive and the measured spectrum of each adhesive. Solid lines express Correlation Coefficient within the same sample. Dot lines express Correlation Coefficient with the other samples.

The estimated distributions of each sample using the aforementioned correlation coefficient analysis are shown in Fig. 6(a). The distributions of all samples can be estimated quite precisely. Especially for Samples 1 and 3, the pixels were markedly brighter than where other adhesives were present, and there was a high discrimination ability. For Samples 2 and 4, their spectra were similar and both pixels were bright, but Samples 2 and 4 were still identifiable. Therefore, it can be said that even a slight spectral difference can be properly identified.

Figure 6(b) shows histograms of the frequency of appearance of the correlation coefficient between the representative spectrum and the spectrum of each pixel for each adhesive. Regarding the representative spectra of all of the adhesives, the correlation coefficients with the spectrum of the same adhesive showed higher values, which were roughly 0.9 or more. On the other hand, the correlation coefficients with the spectra of different adhesives are distributed frequently in the vicinity of 0.6 in the case of Sample 1, less than 0.4 for Sample 3, in the vicinity of 0.5 and 0.8 for Sample 2, and in the vicinity of 0.6 and 0.8 for Sample 4. From the above results, it was clarified that when examining the distribution of adhesives using this method, a correlation coefficient of about 0.9 was a suitable threshold to determine whether the same adhesive was present or not.

## Discussion

We have constructed a mid-infrared hyperspectral imaging device that can measure macroscopic-sized samples using inexpensive and easy-to-handle bolometer cameras and imaging-type interferometers with high light-utilisation efficiency. By measuring various samples, it was found that hyperspectral images can be successfully measured when an organic substance, such as an adhesive, was applied as a thin layer on a highly reflective object such as an aluminium plate. Samples 1 and 4 and Samples 2 and 3 used in this experiment could not be distinguished by the conventional visible/near-infrared spectroscopic/fluorescent photography method in the wavelength range 0.4–1.0 μm, but our newly developed method could identify all of the adhesives. Therefore, mid-infrared hyperspectral imaging is expected to become a new method to identify samples that cannot be identified by conventional methods.

Unfortunately, mid-infrared hyperspectral imaging could not be successfully measured if the adhesive layer was too thick, or if an adhesive was placed on a light-scattering surface such as paper. However, in the case of paper with a relatively high reflectance such as glossy paper, the measurement could be performed successfully. It is expected that if the experimental system can be further improved in the future and organic matter on scattering surfaces can be measured, the range of applications will be expanded. Although we are studying this method for forensic science and cultural properties science, it is expected that this method can be applied to discover defects in products on factory production lines or to visualise the state of deterioration of infrastructure.

The shape of the representative spectra (Fig. 5(a)) does not necessarily agree exactly with the spectra measured by FTIR (Fig. 5(b)), but the absorption peaks usually coincide to some extent. The reason for the difference in shape of spectra is considered to be as follows. While the commercially available FTIR measures 2.5–14 μm, the instrument of this study measures 8–14 μm. Therefore, the detector used in this study has low sensitivity near the wavelength of 8 and 14 μm. In addition, although the measurement result of this study is the average value of the spectra of a wide area (about 1 cm × 1 cm square) of the sample, results of the commercial FTIR are the average value of the spectra of the region of 25 μm × 25 μm.

The results of this method include other components such as reflection components on the adhesive surface in addition to the absorption spectrum of the adhesive^3^. Furthermore, in this experimental system, since the light was obliquely irradiated only from one direction, there was a large unevenness in the amount of light on the sample. Despite the severe conditions described above, measurements made by this device, together with analysis using the correlation coefficients, were able to accurately estimate the position of each sample. This means that the spectrum of each point can be accurately measured, and that the spectrum can be measured in the same way regardless of spatial position.

As shown in Fig. 6(b), for all the representative spectra of the adhesives, the correlation coefficient with the spectrum of the same adhesive exhibited higher values than the correlation coefficients with the spectra of different adhesives. Therefore, it was found that adhesives can be distinguished by setting a threshold value for the correlation coefficient. In the cases of Samples 2 and 4, some of the spectra of different adhesives had relatively high correlation coefficients. This is because both samples are polyvinyl based adhesives and their spectra are similar. In this study, the spectrum over the whole wavelength band of 8–14 µm was used for analysis. However, it is expected that better discrimination ability and better results will be obtained if the analysis is performed using only a wavelength band in which spectral differences appear between samples.

We consider the interference due to the thickness of the sample as follows. We were careful not to paint the samples too thick on the aluminium plate. This is because if the sample is too thick, infrared light will not be reflected at all and the spectra cannot be measured. But the thickness of the sample was not controlled. Therefore even the same sample has different thickness depending on location. When interference occurs, the reflectance is considered to become high at a specific band of wavelength. However, in the calculation of the representative spectra, since the spectra of a wide region (the spectra of different sample thickness) were averaged, the influence of interference, even if it exists, is thought to be cancelled out. In addition, as the results of the correlation coefficient analyses show, the correlation with the representative spectrum did not change even if the sample thickness was different, so it is considered that the influence of interference did not exist or was not large.

## Conclusion

We have constructed a mid-infrared hyperspectral imaging device that can measure macroscopic-sized samples using inexpensive and easy-to-handle bolometer cameras and imaging interferometers with high light-utilisation efficiency. It was found that mid-infrared hyperspectral images can be successfully measured when organic materials such as adhesives are thinly painted onto highly reflective objects such as aluminium plates. In addition, we could not only measure the spectrum of each single point accurately but also measure the spectrum in the same way regardless of the spatial position. Using this method, we can accurately measure the spatial distribution of different types of adhesive that cannot be discriminated by conventional visible/near-infrared spectroscopic/fluorescent photography with wavelengths in the range 0.4–1.0 µm. Therefore, our approach is expected to become a new method for inspecting samples that cannot be identified by conventional methods.
